# Characterization of the Phenotypic and Genotypic Antibiotic Resistance Markers in *Escherichia coli* (*E. coli*) Associated With Diabetes and Nondiabetic Patients

**DOI:** 10.1155/ijm/3694023

**Published:** 2025-01-31

**Authors:** David Nana Adjei, Thomas Stuart Mughogho, Olu-Taiwo Michael, Sarah Saidu, Gloria Amegatcher, Akua Obeng Forson

**Affiliations:** Department of Medical Laboratory Science, School of Biomedical and Allied Health Sciences, College of Health Sciences, University of Ghana, Legon, Accra, Ghana

**Keywords:** antibiotic resistance, diabetes patients, *Escherichia coli* (*E. coli*), Ghana, nondiabetic patients

## Abstract

**Introduction:** Individuals with diabetes are more susceptible to urinary tract infections (UTIs) than those without the disease. This study aimed to determine the phenotypic and genotypic antibiotic resistance profiles of *Escherichia coli* in diabetic and nondiabetic patients.

**Methodology:** A total of 374 clean-catch midstream urine specimens were screened for uropathogens, and antibiogram analysis was done on *E. coli* isolates by the Kirby–Bauer disc diffusion method, followed by phenotypic confirmation of extended spectrum beta-lactamase (ESBL) production. In addition, polymerase chain reaction (PCR) assays were carried out to determine ESBL genotypes.

**Result:** Overall, we observed UTIs prevalence of 19.8% and 10.7% in diabetic and nondiabetic patients. Females exhibited higher UTI prevalence than males in both groups ([71.8% and 28.2%] vs. [85% and 15%]) (*p* < 0.0001). Among women with and without diabetes, the age groups of 55–64 and 25–34 years showed the highest prevalence of UTIs (25.6% vs. 40%). The most prevalent uropathogen was *E. coli* (62.2% vs. 75%); multidrug-resistant (MDR) *E. coli* was (61% vs. 33.3%) and ESBL–*E. coli* was (34.8% and 20%) in diabetic and nondiabetic patients, respectively. The most common ESBL-mediated gene was *bla*CTX-M (64.3%) with multiple ESBL genes in some *E. coli* isolates. High-level resistance was observed for ampicillin (91.2%), cefuroxime (96.7%), ciprofloxacin (44.9%), and trimethoprim (59.4%), and low-level resistance was observed for gentamicin (18.7%), ceftriaxone (20.9%), and nitrofurantoin (19.8%). There was no significant difference between antibiotic resistance in diabetic and nondiabetic patients (*p* > 0.05).

**Conclusion:** We observed *bla*CTX-M as the most common ESBL genotype, in combination with other ESBL genes present in some *E. coli* isolates. Nitrofurantoin and ceftriaxone antibiotics were efficacious. Appropriate prescription of antibiotic therapy, and the prevention of transmission of resistant genes in the context of public health can be facilitated by routine monitoring of the resistance profiles and ESBL markers in patients with and without diabetes.

## 1. Introduction

In Africa, diabetes is one of the leading causes of death across all demographic groups [1]. It was predicted that 463 million people worldwide had diabetes in 2019, with type 2 diabetes mellitus making up more than 90% of cases, and by 2030, this figure is projected to increase to 552 million [1]. This is one of the highest predicted increases among all regions of the world, and the prevalence and burden of diabetes mellitus is growing swiftly in sub-Saharan Africa [1]. In Ghana, the overall prevalence of diabetes among adults is projected to be 6.46% [2].

Urinary tract infections (UTIs) are a major public health concern for both diabetics and nondiabetics, with diabetics being more predisposed to UTIs [3]. While UTIs are frequently seen in individuals with diabetes, their occurrence has been linked to either individual risk factors, local factors, or a combination of both [4]. The majority of UTIs in diabetic people are comparatively asymptomatic, but if treated carelessly, they can cause kidney damage and renal failure [4–6]. Several studies have reported a high rate of *Escherichia coli* and *Klebsiella* spp. among UTIs in type 2 diabetic patients [7, 8]. Globally, UTIs and its related complications are estimated to reach 150 million deaths per year [9]. In sub-Saharan Africa, a 20%–39% prevalence of UTIs in persons with diabetes has been reported, and the incidence has shown to be higher in people with diabetes than in those without diabetes [2, 10–14]. As antimicrobial resistance (AMR) increases, managing UTIs can become a significant challenge for both diabetic and nondiabetic individuals. The published literature has showed that an increase in bacterial resistance to commonly used antibiotics could lead to about 10 million deaths globally each year, as estimated by O'Neil et al. [15]. Despite this, the prevalence of uropathogenic infections among diabetic patients with UTIs is rising across different regions [16]. In sub-Saharan Africa, the majority of public laboratories do not evaluate extended spectrum beta-lactamase (ESBL) phenotypes in diabetic patients with UTIs, and data on genotypic ESBL determinants are scarce. Furthermore, very limited studies have been conducted to generate relevant data on the phenotypic and genotypic AMR markers of uropathogens associated with diabetes and nondiabetic patients. This study aimed to determine the phenotypic profiles and genotypic ESBL resistance markers of *E. coli* associated with diabetes and nondiabetic patients. This research will contribute to formulate targeted strategies to mitigate the effects of multidrug-resistant (MDR) pathogens in diabetic individuals suffering from UTIs.

## 2. Materials and Methods

### 2.1. Study Design and Area

The study was a descriptive cross-sectional study carried out at the Mamprobi Hospital in the Greater Accra region of Ghana from 2020-2021. Geographically, the hospital is located at the capital city of Ghana with “Accra” usually referred to as the Accra metropolitan area [17]. Mamprobi Hospital had all the facilities and departments required for a primary healthcare system including outpatient departments and laboratories as well as a dedicated diabetic clinic where people living with diabetes were seen regularly. Mamprobi Hospital is also only a short drive away from Ghana's premier referral and tertiary care hospital, the Korle-Bu Teaching Hospital (KBTH).

### 2.2. Sample Collection and Processing

Each prospective patient was directed on how to provide clean-catch mid-stream urine of 10–20 mL in a sterile wide-mouth urine container. All collected samples were labeled and transported within 2 h of collection to the laboratory for culture.

### 2.3. Data Collection

Data on sociodemographic and other information including clinical symptoms, previous antibiotic use, and length of antibiotic usage were collected using a standardized questionnaire.

### 2.4. Isolation and Identification of Uropathogens

Employing the semi-quantitative technique, a calibrated sterile loop of 0.001 mL urine sample was streaked on cysteine–lactose electrolyte deficient (CLED) medium agar plates (Oxoid, UK). The plates were then incubated at 37°C for 18–24 h. Bacterial cells of uniform shape and size of colony counts (≥ 10^5^ cfu/mL) were suggestive of significant bacteriuria and infection. Discrete colonies from significant agar plates were phenotypically characterized as per standard biochemical tests [18].

### 2.5. Antibiotic Susceptible Testing

Confirmed uropathogenic isolates were subjected to antibiotic susceptibility testing by the Kirby–Bauer disc diffusion method as per guidelines of the Clinical and Laboratory Standard Institute [19]. Briefly, one to two isolated colonies were picked from an overnight culture and suspended in 5 mL of sterile saline solution with turbidity compared to 0.5 McFarland standard. Using a sterile cotton swab, the suspension was evenly streaked on Mueller–Hinton agar plate (Oxoid, UK) and incubated at 37°C for 18–24 h. The following 10 commercial antibiotics were employed: ampicillin (10 μg), cefotaxime (30 μg), ciprofloxacin (5 μg), gentamicin (5 μg), cefuroxime (30 μg), ceftazidime (30 μg), ceftriaxone (30 μg), gentamicin (10 μg), trimethoprim (30 μg), and nitrofurantoin (300 μg) (Oxoid, UK). *Klebsiella pneumoniae* ATCC 700603 and *E. coli* ATCC 25922 were used as control strains to assist in the evaluation of the performance of the test. According to the international standard definitions for acquired resistance, and relative to the panel of antibiotics tested, an MDR phenotype was defined as in vitro non-susceptibility to ≥ 1 agent in ≥ 3 antimicrobial categories [20].

### 2.6. Phenotypic Confirmation of ESBL by Combination Disk Diffusion Test

ESBL-producing isolates were confirmed phenotypically through the combination disk diffusion test as briefly described by CLSI, [19]. ESBL-producing isolates were based on an increase in zone diameter of ≥ 5 mm for either cefotaxime or ceftazidime in combination with clavulanic acid, when compared with either of the cephalosporins alone. Control strains of *E. coli* ATCC 25922 and *K. pneumoniae* ATCC 700603 served as negative and positive controls, respectively [19].

### 2.7. Genotypic Detection of Resistant Genes

Isolates with ESBL phenotypes were confirmed with multiplex polymerase chain reaction (PCR) to determine the presence of ESBL genes.

### 2.8. DNA Extraction

Extraction of DNA was done using the crude boiling method. For each isolate, 10 μL loopful of pure bacterial isolate were inoculated into sterile 2 mL Eppendorf tubes containing 200 μL of nuclease-free water. This mixture was vortexed and boiled in a heat block for 10 min. A heat shock was then applied to the inoculum by freezing it for another 10 min to further break the cells of the bacteria for the DNA to be extracted [21]. The preparation was centrifuged at 1300 rpm for 10 min, and the supernatant was collected into a clean 1.2-mL Eppendorf tube and stored in a −20°C freezer.

### 2.9. PCR

For PCR assays, each reaction mixture comprised of a 25-μL final reaction volume with 12.5 μL of 2 x DreamTaq Green PCR Master Mix, 4.5 μL of the primer mix (Table 1), 6 μL of molecular grade nuclease-free water, and 2 μL of DNA template. A negative control was set with the master mix and water as the template. A multiplex PCR reaction was used to determine the presence of *bla*TEM, *bla*SHV, and *bla*CTX-M as described by Panda et al. [22]. *K. pneumoniae* ATCC 700603 was used as positive control. Thermocycling was performed at an initial denaturation at 94°C for 5 min, followed by 30 cycles of denaturation at 94°C for 1 min, primer annealing at 57°C for 1 min, extension at 72°C for 1 min, and a final elongation temperature at 72°C for 10 min. All PCR amplicons were analyzed by horizontal gel-electrophoresis in a 2% (weight/volume) agarose gel using Tris/Acetate/EDTA 50 x concentrate buffer.

## 3. Results

### 3.1. Demographics of Participants

A total of 374 participants were recruited in this study, with 187 participants for each group (diabetic and nondiabetic) patients. Most participants were females in both groups of patients (Table 2). The mean ages were 56 years (age range; 18–94 years) in diabetics and nondiabetics. The average age groups were 55–64 and 65–74 for females and males, respectively (Table 2).

### 3.2. Distribution of Positive Culture in Diabetic and Nondiabetic Patients

In both diabetes and nondiabetic individuals, the majority of isolates came from female participants rather than men, (71.8%, *n* = 28) and (85% *n* = 17) (Table 3). In the nondiabetic group, females aged between 25 and 34 years had the highest incidence of UTIs (40%, *n* = 8), and the least affected in the nondiabetic group were age groups above 65 years. In the diabetic group, females aged 55–64 had the highest incidence of UTIs (25.6%, *n* = 10).

### 3.3. Distribution of Uropathogenic Isolates

A total of 59 bacterial uropathogens were isolated from positive urine culture (≥ 10^5^ CFU/mL) representing a 15.8% (59/374) overall prevalence, with a specific prevalence of 19.8% and 10.7% in type 2 diabetics and nondiabetics, respectively (Table 4). *E. coli* was the common isolated uropathogen in both diabetics and nondiabetics (62.2% vs. 75%), followed by *Klebsiella* spp. (10.8% vs. 15%), *Enterobacter* spp. (8.1% vs. 5%), and *Morganella* spp. (8.1% vs. 0%). The differences in the distribution of *E. coli*, *Klebsiella* spp. and *Enterobacter* spp. isolates were not statistically significant (*p* > 0.05) (Table 4).

### 3.4. Prevalence of Resistance Profile

*E. coli* isolates recovered from diabetic and nondiabetic patients exhibited high-level resistance to ampicillin (95.7% vs. 86.7%), cefuroxime (100% vs. 93.4%), and trimethoprim (65.4% vs. 60%), respectively (Table 5). Moderate resistance to ciprofloxacin (56.4% vs. 52%) and nalidixic acid (43,8% vs. 26.7%) was also observed in diabetic and nondiabetic patients. Overall, there was no significant difference between resistance to antibiotics in diabetic and nondiabetic patients ([*p* > 0.05] Table 5). *E. coli* isolates among diabetic and nondiabetic patients revealed MDR prevalence of 61% (14/23) and 33.3% (5/15) with no significant difference ([*p* > 0.05] Table 6).

### 3.5. Prevalence of ESBL–*E. coli* in Diabetics and Nondiabetics

The combined disc diffusion test showed that 39.1% (9/23) and 40.0% (6/15) of *E. coli* isolates from diabetic and nondiabetic were phenotypically positive for ESBL production. The total distribution of the phenotypically ESBL-producing *E. coli *was not statistically different between type 2 diabetes and nondiabetics (39.5%).(Table 7). The majority of ESBL-positive *E. coli* isolates were recovered from females in both type 2 diabetic 13.0% (3/23) and nondiabetic 13.3% (2/15) patients. In nondiabetics, ESBL-positive *E. coli* was higher in the younger age groups (under 34 years groups), while in the diabetics, ESBL-positive *E. coli* were higher in the older groups (55–64 years).

### 3.6. Genotypic Characterization

Of the 15 ESBL-producing *E. coli*, 14 harbored an ESBL genes: *bla*TEM (3 *E. coli*), *bla*SHV (1 *E. coli*), *bla*CTX-M (4 *E. coli*), *bla*(CTXM + TEM) (2 *E. coli*), *bla*(CTXM + SHV) (1 *E. coli*), and *bla*(CTXM + SHV + TEM) (2 *E. coli*). *bla*CTX-M was the most predominant ESBL gene (*n* = 9, 64.3%) and 1 *E. coli* carried *bla*(TEM).

## 4. Discussion

Several research studies have demonstrated that UTI remains one of the most prevalent infectious diseases associated with diabetic patients in the community and hospital settings [23–25]. This study report for the first time varying phenotypic and genotypic antibiotic resistance profiles of *E. coli* from diabetic and nondiabetic patients in Ghana. Limited routine detection of AMR uropathogenic bacteria in UTI diabetic patients makes this study's findings very important for public health prevention, awareness, and proactive therapeutic management of diabetic UTI patients.

In this study, an overall UTI prevalence of 19.8% was observed among diabetic patients, as compared to 10.7% in nondiabetic patients. Previous studies in Ethiopia, Sudan, and Saudi Arabia have reported a 19.5%–25.3% prevalence [26–29]. In contrast to these findings, higher prevalence of 38.3%–67.5% have been reported among the diabetic patients in Ghana, Cameroun, Somalia, Kuwait, and Malaysia [10, 30–32]. An earlier study by Saleem and Daniel [33] reported UTI prevalence of 56.4% and 43.6% in diabetic patients and nondiabetic patients with a poor socioeconomic status. Diverse factors may be attributed to UTI in diabetic patients, including genetic susceptibility and suppressed or damaged immune response [33]. Furthermore, the varying prevalence among these studies may be due to diverse sociodemographic and cultural settings [34, 35].

In this study, 71.8% of diabetic female patients were UTI culture positive as compared to 85% among nondiabetic female patients. This finding is similar with the 73.5% prevalence reported in Cameroun [36]. Several studies in Ghana, Portugal, China, Iran, and Bangladesh have documented higher UTI incidence in females than in males [10, 34, 37–39]. This is primarily due to the short urethra, lack of prostatic secretion, pregnancy, and frequent urinary tract contamination with fecal flora [35, 40]. In this study, diabetic patients exhibited UTI positivity of 25.6% among the 55–64 years age category. A study in Senegal by Barrya et al. [41] have likewise reported UTI positivity of 46.6% among females aged ≥ 60 years. In Cameroun, Signing et al. [36] reported UTI positivity of 50.6% among females aged ≥ 60 years. These findings may be associated with suppressed immune response and age, which can be escalated by the occurrence of severe and debilitating forms of UTI [36, 42]. In the nondiabetic patients, UTI positivity was 40% in the females in the age group 25–35 years. Similar findings were reported in Indonesia by Rahimi et al. [43]. An earlier study in Nepal by Yardav and Pradash [44] reported a higher UTI among females (70.45%) in the age group 21–40 years among nondiabetic patients. This finding may be attributed to the sexually active and productive age of females with the risk of recurrent UTI [43].

The prevalent uropathogens observed among diabetic and nondiabetic patients in this study were *E. coli* (62.2% vs. 75%), followed by *Klebsiella* spp. (21.6% vs. 20%). Among UTI-causing bacteria, about 80%–85% were Gram-negative bacilli, with *E. coli* being the primary etiological agent for 75.5%–87% of UTI cases. Previous studies have reported a 55.1%–66.2% prevalence in India, Ghana, China, and Nepal [34, 44–46]. However, the prevalence is lower than 93% reported in Somalia [31]. The variations in prevalence for *E. coli* may be due to diversity in the study design and sample size [45]. Furthermore, *E. coli* strains harbor a broad repertoire of virulence attributes that facilitate their uroepithelial adherence, such as the colonization of phenotypic receptors in the urogenital mucosa with adhesives, pili, fimbriae, and P-1 [47].

In the last decades, the increasing prevalence of AMR has become a huge public health challenge in the general population and in particular diabetic patients [48–50]. In the present study, *E. coli* isolates in diabetic patients exhibited high levels of resistance to diverse categories of antibiotics. Multi-drug resistant* E. coli* among diabetic patients was 61%, as compared to 33.3% in the nondiabetic patients. A study in China by He et al. [34] reported MDR prevalence of 50% among diabetic patients. Even higher than our finding, an elevated prevalence of MDR *E. coli* has been reported in Nepal (78.6%) and Turkey (68.7%) [44, 51]. Previous studies in Asia have reported MDR *E. coli* prevalence of 52%–81% [52, 53]. More recently, a study with diabetic patients from different parts of Ethiopia have reported MDR prevalence of 59.8%–71.7% [54]. The variation in the prevalence of MDR *E. coli* strains might be due to racial differences as well as indiscriminate and inappropriate use of antibiotics for asymptomatic or moderate symptomatic UTI in some geographical settings [34].

ESBL-producing *E. coli* strains often exhibit an MDR phenotype, which include coresistance to aminoglycosides and fluoroquinolones [55]. In this study, the prevalence of ESBL among diabetic and nondiabetic patient were 39.1% (9/23) and 40% (6/15). Similar findings of 48.5% prevalence have been reported in China [53]. In Algeria, a lower ESBL prevalence of 32.2% has been reported by Zenati et al. [56]. Studies elsewhere have observed a higher prevalence in diabetic patients compared to nondiabetics [57, 58]. The published literature shows that ESBL-mediated genes are associated with resistance to second and third generation cephalosporins. Moreover, our study observed moderate levels of resistance to ciprofloxacin (56.4%). This was higher than 21.4%–32.7% reported in previous studies from Ghana, Algeria, and Nepal [44, 45, 56]. Our study's finding is also higher than the 30%–34% reported in Kuwait, Nepal, and Ethiopia [32, 44, 55].

Diabetic *E. coli* strains exhibited moderate levels of resistance to third generation cephalosporins (ceftazidime [55%], cefotaxime [43.5%], and ceftriaxone [21.7%]). Similar moderate resistance of 28.6%–55.6% to cefotaxime has been reported in India, Egypt, Ghana, and Kuwait [32, 45, 55, 57]. In contrast to our study, Ifediora et al.'s [59] study in Nigeria reported 0% to ceftriaxone. The variation of *E. coli* resistance to cephalosporin in some regions may be attributed to ESBLs induced by selective pressure from broad-spectrum antibiotic therapy [60].

For gentamicin, a member of the aminoglycoside class of antibiotics, a low resistance of 17.4% was found in this study. Similar findings of 14.3% and 12.5% have been reported in Ghana and Ethiopia [45, 61]. By comparison, higher levels of resistance of 31.5%–72.1% have been reported in India, Nepal, and Ethiopia [14, 44, 57]. The low usage of this antibiotic, largely in the form of combination therapy, may have promoted the low resistance level found in this study. Antibiotics such as nitrofurantoin also exhibited a low resistance of 13.0%. A similar finding of 11.8% has been reported in Bangladesh [62]. Furthermore, findings from other localities have reported the efficacy of nitrofurantoin treatment against uropathogens [10, 32]. With respect to trimethoprim–sulfamethoxazole, this study observed a resistance level of 65.5%. A lower resistance of 42.8% and 48% has previously been reported in Ghana and Kuwait [32, 45]. A study in Turkey by Bagir et al. [51] reported a higher level of resistance (81.8%) to trimethoprim–sulfamethoxazole [51]. The high level of resistance found for trimethoprim–sulfamethoxazole in our study is concerning; it may be due to inappropriate and indiscriminate use of these antibiotics [10].

## 5. Conclusion

The present study revealed UTI prevalence of 19.8% and 10.7% in diabetic and nondiabetic patients, with *E. coli* being the most prevalent uropathogen. *E. coli* exhibited MDR features of 61% and 33.3% in diabetic and nondiabetic patients, with no significant difference among tested antibiotics. The most prevalent ESBL gene was *bla*CTX-M, but the occurrence of *E. coli* with multiple ESBL genotypes was also detected. Regular monitoring of resistance profiles in uropathogens and addressing challenges of treating ESBL infections in diabetic and nondiabetic patients is crucial for maintaining public health.

## Figures and Tables

**Table 1:** List of primers used for multiplex PCR.

Genes ESBLS	Primers (5′-3′)	Size (bp)
*bla*SHV	GTCAGCGAAAAACACCTTGCC	383
	GTCTTATCGGCGATAAACCAG	

*bla*TEM	GAGACAATAACCCTGGTAAAT	460
	AGAAGTAAGTTGGCAGCAGTG	

*bla*CTX-M	GAAGGTCATCAAGAAGGTGCG	520
	GCATTGCCACGCTTTTCATAG	

**Table 2:** Demography of participants.

Age range	Diabetics		Nondiabetics	
	Female	Male	Female	Male
15–24	6 (4.22)	1 (2.22)	26 (17.1)	3 (8.8)
25–34	16 (11.3)	2 (4.44)	42 (27.6)	8 (22.9)
35–44	20 (14.1)	6 (13.3)	26 (17.1)	3 (8.8)
45–54	8 (5.6)	10 (22.2)	17 (11.1)	4 (11.4)
55–64	20 (14.1)	5 (11.1)	15 (9.9)	8 (22.9)
65–74	47 (33.1)	15 (10.5)	17 (11.1)	7 (20.0)
75–84	24 (16.9)	6 (13.3)	8 (5.2)	2 (5.7)
85–94	1 (0.70)	0 (0)	5 (3.29)	0 (0)

Total	142 (37.9)	45 (12.03)	152 (40.6)	35 (9.4)

**Table 3:** Distribution of positive culture in diabetic and nondiabetic participants.

Age range	Diabetics		Nondiabetics	
	Female	Male	Female	Male
15–24	—	—	4 (20)	—
25–34	3 (7.7)	—	8 (40)	1 (5)
35–44	5 (12.8)	—	3 (15)	1 (5)
45–54	5 (12.8)	3 (7.7)	—	1 (5)
55–64	10 (25.6)	4 (10.2)	1 (5)	—
65–74	5 (12.8)	4 (10.2)	—	—
75–84	—	—	1 (5)	—
85–94	—	—	—	—

Total	28 (71.8%)	11 (28.2%)	17 (85%)	3 (15%)

**Table 4:** Distribution of uropathogens in the participants.

Uropathogens	Diabetics	Nondiabetics	*p* value
	No. (%)	No. (%)	
*Escherichia coli*	23 (62.2)	15 (75)	0.205
*Klebsiella oxytoca*	5 (10.8)	3 (15.0)	0.43
*Klebsiella pneumoniae*	5 (10.8)	1 (5.0)	0.125
*Morganella* spp.	3 (8.1)	—	0.001
*Enterobacter* spp.	3 (8.1)	1 (5.0)	0.202

Total	39 (100)	20 (100)	

**Table 5:** Resistant patterns of *Escherichia coli* isolates.

Antibiotics	Diabetics (no. = 23)	Nondiabetics (no. = 15)	*p* value
AMP	22 (95.7)	13 (86.7)	0.166
CAZ	12 (55.0)	5 (33.3)	0.207
CTX	11 (47.8)	8 (53.3)	0.446
CXM	23 (100)	14 (93.4)	0.17
CRO	5 (21.7)	3 (20.0)	0.477
CIP	13 (56.4)	5 (33.3)	0.189
GEN	4 (17.4)	3 (20.0)	0.465
NAL	10 (43.8)	4 (26.7)	0.276
NIT	3 (13.0)	4 (26.7)	0.329
SXT	15 (65.4)	8 (53.3)	0.285
MDR	14 (61.0)	5 (33.3)	0.143

Abbreviations: AMP, ampicillin; CAZ, ceftazidime; CIP, ciprofloxacin; CRO, ceftriaxone; CTX, cefotaxime; GEN, gentamicin; NAL, nalidixic acid; NIT, nitrofurantoin; SXT, trimethoprim.

**Table 6:** Multidrug-resistant profile of *Escherichia coli* in diabetic and nondiabetic patients.

Multidrug-resistant profile of *E. coli* in diabetic patient	
AMP + CAZ + CTX + CXM + CRO + CIP + GEN + NAL + NIT + SXT	3
AMP + CAZ + CTX + CXM + CRO + CIP + NAL + SXT	2
AMP + CAZ + CTX + CXM + CIP + NAL + SXT	5
AMP + CAZ + CTX + CXM + CIP + SXT	2
AMP + CXM + CIP + SXT	1
AMP + CXM + SXT	1

Total	14

**Multidrug-resistant profile of *E. coli* in nondiabetic patient**	

AMP + CAZ + CTX + CXM + CRO + CIP + GEN + NAL + NIT + SXT	3
AMP + CAZ + CTX + CXM + CRO + CIP + NAL + SXT	1
AMP + CAZ + CTX + CXM + CIP + SXT	1

Total	5

Abbreviations: AMP, ampicillin; CAZ, ceftazidime; CIP, ciprofloxacin; CRO, ceftriaxone; CTX, cefotaxime; GEN, gentamicin; NAL, nalidixic acid; NIT, nitrofurantoin; SXT, trimethoprim.

**Table 7:** Distribution of ESBL positive *E. coli* by age and gender in diabetic and nondiabetic patients.

	Diabetic patients (no. = 23)		Nondiabetic patients (no. = 15)	
Total no. of ESBL-*E. coli* (*n* [%])	9 (39.1)		6 (40.0)	
Gender				
Male	3 (13.0)		2 (13.3)	
Female	6 (26.1)		4 (26.7)	

**Age group (yrs)**	**Female**	**Male**	**Female**	**Male**

15–24	—	—	1 (6.67)	—
25–34	1 (4.35)	—	2 (13.3)	1 (6.67)
35–44	—	—	1 (6.6.7)	—
45–54	1 (4.35)	1 (4.35)	—	—
55–64	3 (13.0)	—	—	—
65–74	—	2 (8.7)	—	—
75–84	1 (4.35)	—	—	—

## Data Availability

The data that support the findings of this study are available on request from the corresponding author. The data are not publicly available due to privacy or ethical restrictions.
